# *Shewanella baltica* Ecotypes Have Wide Transcriptional Variation under the Same Growth Conditions

**DOI:** 10.1128/mSphere.00158-16

**Published:** 2016-10-19

**Authors:** W. S. Hambright, Jie Deng, James M. Tiedje, Ingrid Brettar, Jorge L. M. Rodrigues

**Affiliations:** a Department of Cellular and Structural Biology, University of Texas Health Science Center, San Antonio, Texas, USA; b Center for Microbial Ecology, Michigan State University, East Lansing, Michigan, USA; c Department of Vaccinology and Applied Microbiology, Helmholtz Center for Infection Research, Braunschweig, Germany; d Department of Land, Water and Air Resources, University of California, Davis, Davis, California, USA; e Environmental Genomics and Systems Biology Division, Lawrence Berkeley National Laboratory, Berkeley, California, USA; Laboratory of Microbiology, University of Neuchâtel

**Keywords:** ecotype, speciation, transcriptional divergence

## Abstract

In bacterial populations, subtle expressional differences may promote ecological specialization through the formation of distinct ecotypes. In a barrier-free habitat, this process most likely precedes population divergence and may predict speciation events. To examine this, we used four sequenced strains of the bacterium *Shewanella baltica*, OS155, OS185, OS195, and OS223, as models to assess transcriptional variation and ecotype formation within a prokaryotic population. All strains were isolated from different depths throughout a water column of the Baltic Sea, occupying different ecological niches characterized by various abiotic parameters. Although the genome sequences are nearly 100% conserved, when grown in the laboratory under standardized conditions, all strains exhibited different growth rates, suggesting significant expressional variation. Using the Ecotype Simulation algorithm, all strains were considered to be discrete ecotypes when compared to 32 other *S. baltica* strains isolated from the same water column, suggesting ecological divergence. Next, we employed custom microarray slides containing oligonucleotide probes representing the core genome of OS155, OS185, OS195, and OS223 to detect natural transcriptional variation among strains grown under identical conditions. Significant transcriptional variation was noticed among all four strains. Differentially expressed gene profiles seemed to coincide with the metabolic signatures of the environment at the original isolation depth. Transcriptional pattern variations such as the ones highlighted here may be used as indicators of short-term evolution emerging from the formation of bacterial ecotypes.

**IMPORTANCE** Eukaryotic studies have shown considerable transcriptional variation among individuals from the same population. It has been suggested that natural variation in eukaryotic gene expression may have significant evolutionary consequences and may explain large-scale phenotypic divergence of closely related species, such as humans and chimpanzees (M.-C. King and A. C. Wilson, Science 188:107–116, 1975, http://dx.doi.org/10.1126/science.1090005; M. F. Oleksiak, G. A. Churchill, and D. L. Crawford, Nat Genet 32:261–266, 2002, http://dx.doi.org/10.1038/ng983). However, natural variation in gene expression is much less well understood in prokaryotic organisms. In this study, we used four sequenced strains of the marine bacterium *Shewanella baltica* to better understand the natural transcriptional divergence of a stratified prokaryotic population. We found substantial low-magnitude expressional variation among the four *S. baltica* strains cultivated under identical laboratory conditions. Collectively, our results indicate that transcriptional variation is an important factor for ecological speciation.

## Abstract (precis)

Eukaryotic studies have shown considerable transcriptional variation among individuals from the same population. It has been suggested that natural variation in eukaryotic gene expression may have significant evolutionary consequences and may explain large-scale phenotypic divergence of closely related species, such as humans and chimpanzees (M.-C. King and A. C. Wilson, Science 188:107–116, 1975, http://dx.doi.org/10.1126/science.1090005; M. F. Oleksiak, G. A. Churchill, and D. L. Crawford, Nat Genet 32:261–266, 2002, http://dx.doi.org/10.1038/ng983). However, natural variation in gene expression is much less well understood in prokaryotic organisms. In this study, we used four sequenced strains of the marine bacterium *Shewanella baltica* to better understand the natural transcriptional divergence of a stratified prokaryotic population. We found substantial low-magnitude expressional variation among the four *S. baltica* strains cultivated under identical laboratory conditions. Collectively, our results indicate that transcriptional variation is an important factor for ecological speciation.

## INTRODUCTION

The diversity of microorganisms on Earth is staggering, and the relative importance of mechanisms controlling diversification continues to be an important topic. Over the years, evolutionary mechanisms in prokaryotes have been explained as a result of selective pressure being applied on mutations, lateral gene transfer, and shuffling of genetic material for the acquisition of new traits as proteins, enzymes, or entire pathways (1–4). These well-proved mechanisms are responsible for increasing the phenotypic and physiological plasticity of microbial species.

Another suggested possibility for diversification is the natural variation in gene expression (5). Differences in genome-wide expression have been observed for humans (6, 7), mice (8), fish (9), flies (10), and yeast (11). These transcriptional differences were particularly remarkable for closely related species or populations within a species, but their role in adaptive evolution and potential ecological consequences have been difficult to assess.

In nature, where distinct populations of a microbial species can be found at micrometer distances within the community, natural transcriptional variation might be critical for the distribution and maintenance of ecologically different clades of the same microbial species, referred to as ecotypes (12). These distinct groups coexist, exploiting slightly different resources or responding differently to challenging environmental conditions (13–15). Owing to the fluidity of genetic exchanges in microbial systems, fruitful attempts to explain phenotypic differences among ecotypes have been based on cataloging differences in gene content (16–18). Previously, we have demonstrated that environmental isolates of the species *Shewanella*, despite harboring highly similar genomes, showed large proteomic and phenotypic differences under the same growth conditions (19–21), suggesting that transcriptional variation (and regulation) plays an important role in ecological differentiation among strains.

In this study, we asked whether genome-wide transcriptional variation is a contributing factor for ecotype formation within a population of *Shewanella baltica* isolated at different depths of the Baltic Sea, a long-term physicochemically stratified aquatic system. We showed a remarkable natural variation in the expression of genes belonging to the core genome shared among all strains. Our results suggest that low-level changes in gene expression, below threefold, may be a significant contributor to the ecological success of a particular strain within a population.

## RESULTS

### Demarcation of ecotypes.

**FIG 1:**
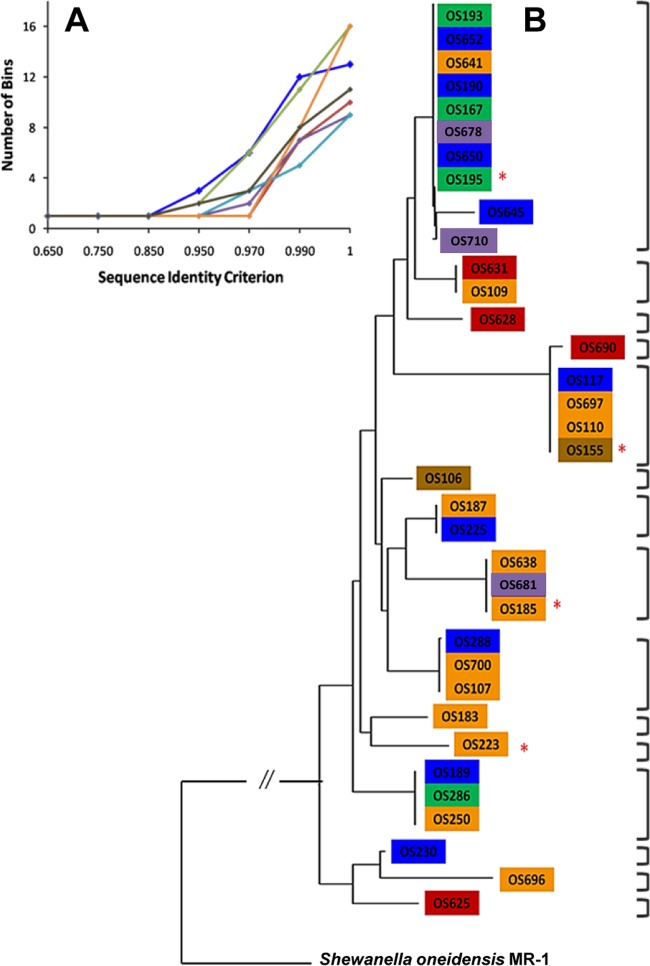
Ecotype simulation (ES) for 36 *Shewanella baltica* strains. (A) Plot including the sequence diversity patterns using 7 different gene sequences as input. Blue, SO0578; red, SO2615; green, SO4702; purple, O2183; teal, SO2706; orange, *gyrB*; brown, SO0625. Simulation was carried out using the 7 genes both exclusively and in concatenated sets yielding consistent demarcations (trees not shown). (B) Representative ES output tree. Red asterisks indicate the four model strains. Brackets indicate ecotypes demarcated by ES. Colors represent depth of isolation, as follows: red, 80 m; brown, 90 m; purple, 110 m; orange, 120 m; blue, 130 m; green, 140 m.

Because *S. baltica* strains are found at various depths of the Gotland Deep water column, a very stratified abiotic environment, we tested whether 36 closely related members of the same species represented different ecotypes. Selected gene sequences were recombination free and used to estimate the rate of ecotype formation and the total number of distinct ecotypes. When gene sequences were used exclusively or as random sets of three concatenated sequences, the number of ecotypes varied from 9 to 16 at a sequence identity criterion of 100% (Fig. 1A). There was a distinct grouping pattern according to water column depth noticed in different output trees. Importantly, strains OS155, OS185, OS195, and OS223 were always placed in distinct clades, regardless of the sequence input used for ecotype demarcation (Fig. 1B).

### Growth assays.

**FIG 2:**
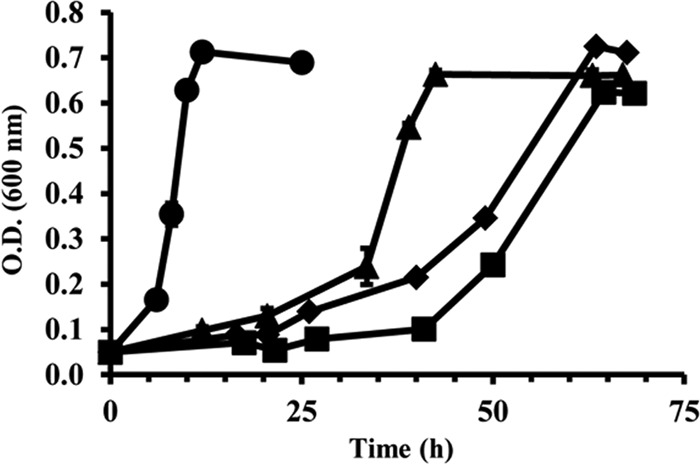
Growth of *S. baltica* strains in defined medium containing glucose as the single carbon source. Growth was measured as the increase in optical density at 600 nm. Circles, OS155; diamonds, OS185; triangles, OS195; squares, OS223. Missing error bars indicate a standard error smaller than the symbol.

*S. baltica* strains OS155, OS185, OS195, and OS223 were isolated from depths ranging from 90 to 140 m with unique redox environments and nutrient availability. In fact, the shewanellae as a whole are known for their incredible redox versatility and are able to utilize a variety of terminal electron acceptors (22). At 140 m, the isolation depth of OS195, the redox environment is unique, with no oxygen, very little nitrate, and relatively more alternative electron acceptors in the form of metal oxides and sulfur-containing compounds. It was thus not surprising that all four *S. baltica* strains had significantly different growth rates and doubling times (*P* < 0.005) when grown in defined medium containing glucose as the sole carbon source (Fig. 2), or maltose for three of the four strains (see Fig. S1 in the supplemental material). OS155, the only strain isolated from a truly oxic zone, was the fastest growing strain in glucose, with an average doubling time and growth rate of 2.05 h and 0.520 h^−1^, respectively. Strain OS195, isolated from the anoxic zone, showed a doubling time of 6.01 h and a growth rate of 0.115 h^−1^, followed by strains OS185 (9.11 h and 0.076 h^−1^) and OS223 (13.3 h and 0.052 h^−1^), both isolated from a transition zone oxic-anoxic interface (oxic-anoxic interface). The aerobic growth characteristics observed for *S. baltica* under these specific culture conditions were generally consistent with those of other studies using closely related shewanellae (23, 24).

10.1128/mSphere.00158-16.4 Figure S1  Growth of *Shewanella baltica* strains in defined medium containing maltose as the single carbon source. Growth was measured as an increase in optical density values at 600 nm. Missing error bars indicate a standard error smaller than the symbol. Circles, OS155; diamonds, OS185; triangles, OS195; squares, OS223. Download Figure S1, TIF file, 0.3 MB. Copyright © 2016 Hambright et al. 2016 Hambright et al. This content is distributed under the terms of the Creative Commons Attribution 4.0 International license.

### Global expression analysis and natural variation among strains.

**FIG 3:**
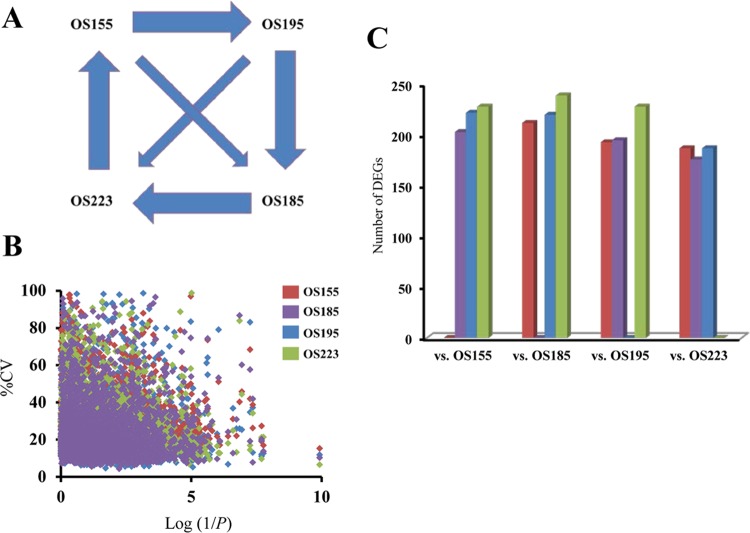
Microarray hybridization scheme and differentially expressed gene (DEG) profiles. Microarray hybridization loop design for all possible pairwise comparisons. Each arrow represents one strain-strain pairwise comparison, including 6 replicate hybridizations. (B) Experimental variation versus significance between strains. Experimental variation per gene (*n* = 3,937) per strain (*n* = 4) reported as %CV against log(1/*P*). *P* values were calculated from ANOVA results. (C) Number of DEGs per strain per pairwise comparison, including unique genes (*n* = 1,179). Unique genes were found at least once in at least one pairwise comparison (repeats not counted).

RNA was isolated during mid-exponential growth in glucose medium from *S. baltica* strains OS155, OS185, OS195, and OS223. mRNA was hybridized to microarrays representing two strains per array for a total of six unique pairwise comparisons (PCs) (Fig. 3A). The quality of the hybridizations and transcriptional profiling was assessed using three metrics: self-to-self slide hybridizations, correlation of percent coefficient of variation (%CV) with significance (*P* value), and quantitative PCR. First, all self-to-self strain comparisons yielded zero differentially expressed genes using the same stringent analysis parameters as nonself comparisons. Second, the %CV for 3,934 genes shared among the four strains was calculated and plotted as the %CV per gene as a function of *P* value. The %CVs were lowest for genes with the highest significance (Fig. 3B). A total of 60.8% of all significant genes showed a %CV error lower than 15%. Third, microarray expression results were confirmed for five randomly selected genes with quantitative PCR (Table 1). Together, these results suggest that the experimental variation did not influence the results and that transcriptional variation between strains was naturally occurring.

**TABLE 1:** Expressional correlation between microarray and quantitative PCR data

Strain comparison	Strain with higher expression	Locus tag of:		Fold change by:		Significance
		Target gene	Housekeeping gene	Microarray	PCR	
OS223 versus OS195	OS195	Shew185_2456	Shew185_0221	9.98	7.48	*P* < 0.001
OS223 versus OS185	OS185	Shew185_2456	Shew185_0221	3.01	5.91	*P* < 0.05
OS185 versus OS195	OS195	Shew185_2456	Shew185_0221	3.31	1.27	*P* < 0.05
OS185 versus OS195	OS185	Shew185_2386	Shew185_0004	1.11	2.30	*P* < 0.01
OS185 versus OS195	OS185	Shew185_1248	Shew185_0004	1.94	4.86	*P* < 0.001

**FIG 4:**
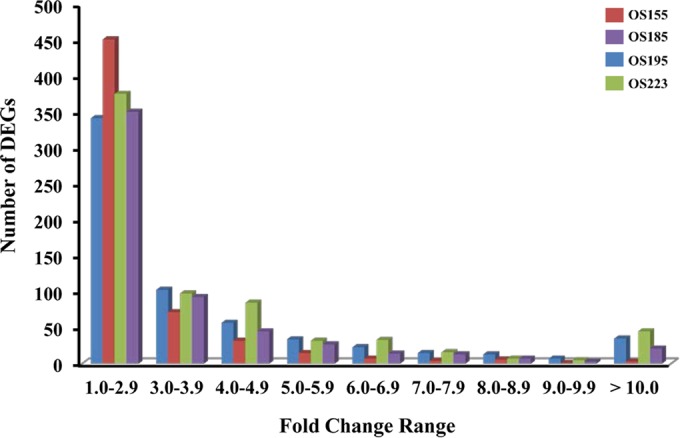
Magnitude of expressional variation among *S. baltica* strains. Global DEG distribution profile indicating fold change range of expressional variation per strain. Genes found to be differentially expressed by multiple strains or in multiple pairwise comparisons were counted for each instance. Individual strain expression is the average of 18 replicates for all three possible pairwise comparisons.

Significant genes using the statistical criterion (*P* < 0.001) were reported as differentially expressed genes (DEGs) irrespective of their direction of expressional change (i.e., up or down). In order to assess transcriptional divergence toward ecotype formation, positive or negative changes in gene expression were considered equally important. We found a total of 1,179 unique DEGs among the four strains (30.0% of the core genome). To better understand the different DEG profiles among strains, we analyzed strain-specific DEGs per PC between strains (Fig. 3C). When particular PCs were analyzed, the number of DEGs varied from 176 to 239 per strain comparison, corresponding to 4.5% to 6.0%, respectively, of the core *S. baltica* genome. Further, DEGs found in either one, two, or all three possible PCs were identified to generate a snapshot profile of strain-specific transcriptional relatedness (Table 2). OS223 and OS195 had the highest number but least diverse set of genes represented, suggesting that both consistently expressed the same DEGs when compared to the other strains. Inversely, OS155, the only strain isolated from truly aerobic conditions, exhibited the highest number of unique DEGs, 361 (Table 2), indicating that OS155 was the most transcriptionally divergent within the group as well as between each PC. The magnitude of the expressional variation was also assessed among the strains (Fig. 4), with 61.0% of DEGs exhibiting less than a threefold change in expression. Most notable was OS155, in which 76.4% of the variance in expression from all pairwise comparisons was less than a threefold change.

**TABLE 2:** Differentially expressed genes per strain by pairwise comparison

Strain	1 PC^a^	2 PC	3 PC	Total (unique)^b^
OS155	153	185	23	361
OS185	84	92	102	278
OS195	68	63	145	276
OS223	44	75	145	264
				
Total	349	415	415	1,179

a PC, pairwise comparison (or unique strain-strain comparison).

b Number of unique genes found when removing redundant occurrences.

We next considered DEGs repeatedly found in every PC among the four strains, as they might highlight the more-essential expressional elements driving ecotype formation. A total of 415 genes were identified, corresponding to 10.6% of the core genome (Table 2). Specifically, strains OS195 and OS223 had the highest number of DEGs, with 145 each (3.7%), followed by strain OS185 with 102 genes (2.6%) and strain OS155 with only 23 genes (0.6%).

### Metabolic reconstruction.

In order to determine potential functional alterations due to transcriptional variation, the set of 415 DEGs found in every PC were examined for general trends in metabolic alterations. First, the DEGs were categorized according to the Clusters of Orthologous Groups (COG) designations (see Fig. S2 in the supplemental material). A total of 22 out of 23 categories were represented by the expression patterns of the four strains. The largest proportion (25.4%) was represented by genes of unknown function (S) and general function prediction (R), followed by three category groups: 9.3% of genes were related to transcription (K), 7.8% to amino acid transport and metabolism (E), and 7.5% to energy production and conversion (C). When organizing DEGs per strain by their respective COG categories, the fastest growing strain (OS155) contained a disproportionate amount of DEGs in categories related to growth. For example, OS155 contained at least 2 times as many DEGs as all other strains combined in categories related to nucleotide transport and metabolism (F), lipid transport and metabolism (I), cellular motility (N), and carbohydrate transport and metabolism (G).

10.1128/mSphere.00158-16.5 Figure S2  COG categories of DEGs found in all pairwise comparisons. Number of DEGs by a factor of less than 3.0 per COG designation. Blue, COG profile of all 415 DEGs; red, COG profile of 415-gene set excluding OS155 DEGs; green, COG profile of only OS155 DEGs. Download Figure S2, TIF file, 1 MB. Copyright © 2016 Hambright et al. 2016 Hambright et al. This content is distributed under the terms of the Creative Commons Attribution 4.0 International license.

Next, metabolic pathways were predicted utilizing the Pathway Tools Omics Viewer (25) using normalized log_2_-transformed intensity values. Here, genes and their associated pathways were deemed significant if there was at least a 3.0-fold difference in intensity value for both increased and decreased gene expression. Both OS155 and OS195, the fastest growing strains, showed upregulation of genes related to the nonoxidative branch of the pentose phosphate pathway, tricarboxylic acid (TCA) cycle, glycolysis, and spermidine biosynthesis. Similar to what was seen in the COG designations versus other strains, OS155 showed higher expression of genes related to aerobic respiration, such as amino acid transport and metabolism, lipid transport, cellular motility, secondary metabolite biosynthesis, and signal transduction mechanisms. Furthermore, OS155 showed decreased expression of membrane transport genes (12 DEGs) compared to their expression in other strains (>20 DEGs).

## DISCUSSION

Studying the forces behind ecological divergence within a natural population is important for understanding the dynamic structure of variation in microbial communities. Here, we have characterized the transcriptional divergence between four ecologically distinct strains of the marine species *Shewanella baltica* isolated from the stratified Gotland Deep water column. All four *S. baltica* strains (OS155, OS185, OS195, and OS223) meet the 16S rRNA gene cutoff value for species identity (19) and share ~96.7% average nucleotide identity for all pairwise genome comparisons (26). Moreover, while OS223 and OS155 do contain genetic rearrangements, OS185 and OS195 share nearly 100% synteny (26). However, the varying abiotic environment of the collection depths suggested these strains may represent ecologically distinct groups, or ecotypes, defined as ecologically distinct units whose diversity is limited by genetic drift or periodic selection (13, 27–31). Ecotypes also often constitute the most recently divergent members of a stratified community (28, 29). Using the sequence-based Ecotype Simulation (ES) algorithm, we found all four *S. baltica* strains to represent unique ecotypes (Fig. 1B). The ES tool also consistently identified several putative ecotypes among other strains present within the same depth, suggesting that depth alone is not the singular determinant of ecotype formation in the *S. baltica* population. In fact, the Gotland Deep water column undergoes turbulent periodic vertical mixing at the oxic-anoxic interface (32). Such mixing would certainly introduce diverse oxidizing targets, a prime environment for the shewanellae given their ability to respire a variety of inorganic substrates (33).

Algorithms like ES are useful metrics to predict ecotypes but rely on genomic sequences, which excludes expressional attributes that may explain divergence and niche partitioning within a community. It is expected that *S. baltica* ecotypes would exhibit different transcriptional profiles *in situ* within the water column. However, we primarily sought to investigate whether the isolates retained divergent and stable transcriptional patterns commensurate with their ecological divergence when grown under identical culture conditions. In this study, we examined transcriptional relatedness among all four ecotypes cultured under identical conditions using minimal defined medium containing a single carbon source. Surprisingly, there were significant differences in growth efficiency among the isolates (Fig. 2), highlighting the metabolic plasticity of the closely related ecotypes. Strain OS155 was the fastest growing of the four ecotypes; it was isolated from an environment containing nitrous oxide and nitrite, as well as the largest amount of dissolved oxygen (34). There is evidence that metabolic plasticity is occurring in our study system, as strain OS155 was found to be the most abundant clone isolated from the Gotland Deep with persistence over time (35, 36). This suggests that OS155 is also competitive in its own environment of isolation.

Our comparative transcriptional analysis using custom microarray slides showed significant expressional divergence among the four ecotypes grown in identical conditions. Two ecotypes were hybridized together using different fluorophores on a single array slide for all possible ecotype-ecotype pairs. Interestingly, each ecotype possessed a cohort of core genes that were consistently differentially expressed in comparison to any other ecotype (Fig. 3C). When ecotypes were compared against one another, sets of differentially expressed genes (DEGs) varied per comparison (Table 2). Furthermore, the degrees of conservation for DEGs among comparisons reflected their ecological divergence. For instance, when compared to the other three ecotypes, OS155 contained a more diverse set of DEGs that changed depending on which ecotype it was compared to (Fig. 3C). It remains to be understood how recently one strain, such as OS155, has diverged from the others.

As a whole, most of the differential expression between ecotypes (61.0%) was less than 3.0-fold (Fig. 4). This phenomenon may be partially explained by stabilizing selection within the community. Studies in eukaryotic systems have suggested that low-magnitude intraspecies transcriptional variation may in fact be governed by stabilizing selection (9, 37–39). As a consequence, this would eliminate large deviations from the expressional mean for the *S. baltica* population. There is evidence in our data set that this is happening with strain OS155, which had the highest frequency of differentially expressed genes (*n* = 293) within the 1.0- to 3.0-fold range (Fig. 4). OS155 seemed to possess a more streamlined expressional profile, favoring small-scale but directed transcriptional regulation leading to its higher growth rate in glucose. This is evidenced by the increase in genes related to glucose oxidation pathways, aerobic energetics, and transcriptional regulation that were differentially expressed in comparison to their expression in the other ecotypes (see Fig. S2 in the supplemental material). This supports the hypothesis that small-scale expressional changes relevant for ecological fitness are stable and measurable.

The transcriptional differences among *S. baltica* ecotypes maybe be explained as a reflection of metabolism within the defined growth conditions. Overall, the expression of genes for similar pathways observed for the faster growing members (OS155 and OS195) indicates a link between growth rate and the emphasis on central metabolic pathways and redox diversity. Furthermore, OS223 and OS185 may prefer nitrate as a terminal electron acceptor, considering that both are thought to be responsible for significant denitrification at their corresponding isolation depths (34). Not having nitrate in the growth medium may have caused the observed shift to alternative respiratory processes for these two ecotypes.

The primary goal of this study was to characterize natural intraspecies transcriptional variation among individuals of a bacterial population as a potential indicator of ecological divergence. Minimal fluctuations in mRNA copy number can vary between individuals and even cells (40) and can reflect natural biological variation unrelated to ecological specialization. Thus, discriminating between meaningful data and noise can be difficult. The observed similarities and differences in transcription profiles among the four *S. baltica* ecotypes reported here are strongly congruent with ecological differentiation (depth), genomic divergence (average nucleotide identity), and pathway relatedness. That is, the most ecologically and phylogenetically similar strains displayed the most similar transcriptomes. For instance, OS223 and OS195 consistently had the largest set of DEGs in comparison to the other two ecotypes (Fig. 3C). This parallels the phylogenetic positioning of strains OS223 and OS195 being more related genomically than the others, namely, strains OS155 and OS185. It is still, however, difficult to predict explicit expressional elements (gene sets or even regulatory networks) responsible for each ecotype’s localized fitness. Such an assay would require conditional responses for each ecotype highlighting their growth success. With the advent of high-throughput sequencing technologies, it will be important in the future to investigate contrasts in gene expression among the strains under multiple other conditions, preferably with parameters that mimic environmental conditions. This will allow a more direct comparison between transcriptome and ecological specification.

Overall, our results suggest that *S. baltica* ecotypes have subtle, stable, and distinct transcriptional signatures under identical growth conditions that may align with ecological divergence. This underscores the proportion and magnitude of natural transcriptional variation among closely related bacterial species. To the best of our knowledge, this study is the first to analyze clade divergence within a population of bacteria using a global transcriptome approach. These findings advance the understanding of intraspecies variation and potential mechanisms responsible for ecological fitness.

## MATERIALS AND METHODS

### Organisms and growth measurements.

*Shewanella baltica* strains OS155, OS185, OS195, and OS223 were originally isolated from the Gotland Deep (57°20′N, 20°03′E) water column as described elsewhere (35). Briefly, seawater samples were collected using sterile champagne bottles on ZoBell bacteriological samplers under different depths (see Table S2 in the supplemental material for abiotic conditions). Specifically, strain OS155 was isolated from Zobell agar incubated aerobically, while strains OS185 and OS195 were isolated from the same medium but incubated anaerobically. Strain OS223 was isolated in diluted liquid nutrient broth (1/10) containing NO_3_^−^. A total of 113 strains were isolated throughout the water column, characterized, and kept as glycerol stocks in the −80°C freezer.

Frozen samples were first cultivated as individual batch cultures using marine broth (Difco, Lawrence, KS) to an optical density at 600 nm (OD_600_) of 0.4. Cells (0.5 ml) were used to inoculate liquid cultures (75 ml) of the Shewanella Federation defined medium, based on modified M1 medium (33), containing 5-mM glucose as the sole carbon source and the following constituents: 7.5 mM NaOH, 28.04 mM NH_4_Cl, 1.34 mM KCl, 4.35 mM NaH_2_PO_4_ ⋅ H_2_O, 30 mM NaCl, 3 mM PIPES [piperazine-*N*,*N*′-bis(2-ethanesulfonic acid)] buffer, trace metals and vitamin solutions as described previously (41), and an amino acid solution containing 2 mg/liter l-glutamic acid, l-arginine, and dl-serine. To minimize maintenance divergence, cells were cultivated from the original frozen stocks to batch culture and then to Shewanella Federation medium, with no subculturing at any time.

### Ecotype demarcation.

Ecotypes were identified with the Ecotype Simulation (ES) algorithm (13). Seven conserved genes were previously sequenced for 36 *S. baltica* strains. All gene sequences were tested and found free of any signs of a previous recombination event prior to their use (13). The sequences used for ES analysis can be found under the following NCBI accession numbers: AAN53658, AAN55644, AAN57661, AAN55227, AAN55734, AAN53703, and ADN96149. Sequences were grouped per gene and aligned with the ClustalX software (42) using different concatenated sets of 2 to 5 *S. baltica* genes combined. Briefly, the ES software demarcates the number of ecotypes (*n*) by estimating rates of ecotype formation (Ω), genetic drift (*d*), and periodic selection (σ), based on the stable ecotype model (30).

### RNA extraction, enrichment, and labeling.

Total RNA was extracted from 5-ml cultures in mid-exponential growth phase (OD_600_ = 0.4) using the RiboPure bacterial RNA kit (Ambion, Inc., Austin, TX). OS155 samples were collected at 7.5 h, followed by OS195 at 35 h, O185 at 50 h, and OS223 at 55 h (Fig. 2). mRNA was isolated from 10 µg total RNA with the MICROB*Express* bacterial mRNA enrichment kit (Ambion, Inc.), followed by amplification and conversion to antisense RNA (aRNA) with the MessageAmp II aRNA amplification kit (Ambion, Inc.). Several studies reported that RNA amplification bias is negligible for related microarray hybridization (43, 44). After ethanol precipitation, aRNA labeling was performed with either Alexa Fluor 555 or Alexa Fluor 647 dye, according to the manufacturer’s instructions (Invitrogen, Inc., Carlsbad, CA). Samples were purified and quality assessed using the Bioanalyzer 2100 (Agilent Technologies, Santa Clara, CA).

### Oligonucleotide microarray design.

Custom glass slide microarrays were generated to represent *S. baltica* strains OS155, OS185, OS195, and OS223 (Mycroarray, Ann Arbor, MI). The genome sizes of the four strains ranged from 5.2 to 5.3 Mb, with 4.3 to 4.5 Mb of shared core genes (see Table S1 in the supplemental material). Each slide contained 30,000 phosphoramidite-synthesized oligonucleotides designed to represent 3,934 genes shared among all four strains (core genome) with 100% sequence identity. Each oligonucleotide was replicated 3 to 7 times onto different regions of the slide (see Data Set S1).

10.1128/mSphere.00158-16.1 Table S1  Genome features and gene contents of sequenced *Shewanella baltica* strains. Download Table S1, DOC file, 0.03 MB. Copyright © 2016 Hambright et al. 2016 Hambright et al. This content is distributed under the terms of the Creative Commons Attribution 4.0 International license.

10.1128/mSphere.00158-16.6 Data Set S1  Microarray slide design and oligonucleotide layout with positive and negative controls. Download Data Set S1, XLS file, 3.1 MB. Copyright © 2016 Hambright et al. 2016 Hambright et al. This content is distributed under the terms of the Creative Commons Attribution 4.0 International license.

### Experimental design and global transcriptional profiling.

Strains OS155, OS185, OS195, and OS223, isolated from the environmentally stratified Gotland Deep, were used for comparative transcriptome profiling (see Table S2 in the supplemental material). Slide hybridizations were performed using three independent biological replicates per strain, with dye swaps for each replicate. Slides were prehybridized with buffer containing 5× SSPE (0.75 M NaCl, 0.05 M NaH_2_PO_4_, 0.005 M EDTA), 1% SDS, and 1-mg/ml acetylated bovine serum albumin at 50°C for 45 min and washed twice with 0.025× SSPE. For each slide, two fluorescently labeled aRNAs from two *S. baltica* strains (2.5 µg each) were resuspended in 60 µl of hybridization buffer containing 1× SSPE, 25% formamide, and 0.05% Tween 20. The mixture was hybridized to the slide at 50°C for 18 h, followed by posthybridization washes with 1× SSPE for 3 min and, subsequently, with 0.1× SSPE at room temperature for 1 min. Slides were scanned at 30-µm resolution using the GenePix 4200A scanner (Axon Instruments, Sunnyvale, CA). Images were analyzed with the GeneSpring GX11 software (Agilent Technologies, Santa Clara, CA), with the median of foreground-background signal intensities imported separately per channel in a one-color format.

10.1128/mSphere.00158-16.2 Table S2  Abiotic parameters within the Gotland Deep water column for each strain isolation depth. Values were extrapolated from Brettar et al. (34). Measurements were recorded during strain isolation in 1986. Download Table S2, DOC file, 0.03 MB. Copyright © 2016 Hambright et al. 2016 Hambright et al. This content is distributed under the terms of the Creative Commons Attribution 4.0 International license.

### Statistical analysis.

The data set, representing 18 hybridizations per strain (including dye swaps), was quantile normalized and log_2_ transformed to a threshold of 1.0 as described in reference 45. Normalized signal intensities were filtered with upper and lower cutoffs of 95% and 10%, respectively, per channel.

In order to test for individual variation, we did not assess the ratios of fluorescent signals but, rather, the intensity of the signal measured as the mean among replicate spots for a specific gene on the slide. Significant differences in gene expression were calculated using one-way analysis of variance (ANOVA) followed by the Benjamini-Hochberg multiple testing correction for all genes. The ANOVA model was selected to distinguish sample standard deviation between-group (SSD_bg_) variation, calculated as

$${\text{SSD}}_{\text{bg}}=\overset{k}{\underset{i=1}{\sum }}\;n_{i}\;{\left(M_{i}-M\right)}^{2}$$

and within-group (SSD_wg_) variation, calculated as

$${\text{SSD}}_{\text{wg}}=\overset{k}{\underset{i=1}{\Sigma }}\;{\text{SSD}}_{i}$$

where the total variance of a data set with *k* groups, of size *n*, with mean (*M*) signal value per gene, can be computed as the sum of the squared deviates (SSD_bg_ + SSD_wg_). The *F* statistic for each gene was calculated with df(*k* − 1) = 3 and with

$$\text{df}\left(\overset{k}{\underset{i=1}{\Sigma }}\;n_{i}-1\right)=18$$

at the *P* <0.001 significance level. *P* values were asymptotically approximated due to the large size of the data set and were normally distributed, as the within-group variation was significantly less than the between-group variation.

Statistical analysis was performed with one-way ANOVA, and type I errors were reduced by applying a significance level of *P* = 0.001 and Benjamini-Hochberg multiple testing correction (46). Only genes whose differences in expression were found to be statistically significant were used for expression profiling, COG profiling, and metabolic pathway analysis. Metabolic pathways were highlighted using the Pathway Tools Omics Viewer (25).

### qPCR.

Differentially expressed genes were selected for quantitative PCR (qPCR) analysis to confirm the microarray data. Specific primers were designed with the OligoAnalyzer software (Integrated DNA Technologies) (see Table S3 in the supplemental material). Amplified aRNA samples (5 µg) were reverse transcribed using the M-MuLV (Moloney murine leukemia virus) *Taq* reverse transcriptase (RT)-PCR kit (New England Biolabs, Ipswich, MA), following the manufacturer’s instructions. qPCR consisted of a reaction mixture with 10 µl SYBR green master mix (BioRad, Hercules, CA), 10 µl containing forward and reverse primers (150 nM each), and 10 ng template. Reactions were performed in triplicate under the following conditions: 5 min of denaturation at 95°C, followed by 40 cycles of denaturation at 95°C for 30 s, primer annealing at 60°C for 30 s, and extension at 72°C for 30 s. Melting curve analysis was used to confirm template specificity and sample purity, and results were analyzed using the cycle threshold (ΔΔ*C_T_*) method (47). The expression levels of two housekeeping genes, *gyrB* and *rpoA*, were used as controls. Statistical significance was calculated with the Student *t* test.

10.1128/mSphere.00158-16.3 Table S3  Primers used for real-time qPCR. Download Table S3, DOC file, 0.03 MB. Copyright © 2016 Hambright et al. 2016 Hambright et al. This content is distributed under the terms of the Creative Commons Attribution 4.0 International license.

### Accession number(s).

Microarray data have been deposited in the Gene Expression Omnibus repository (https://www.ncbi.nlm.nih.gov/geo/) under series accession numbers GSM925307 to GSM925378.
