# Supplementary figures and images for: Shewanella baltica Ecotypes Have Wide Transcriptional Variation under the Same Growth Conditions
Source: mSphere. 2016 Oct 19;1(5):e00158-16. doi: 10.1128/mSphere.00158-16 (PMC5071532; doi:10.1128/mSphere.00158-16)

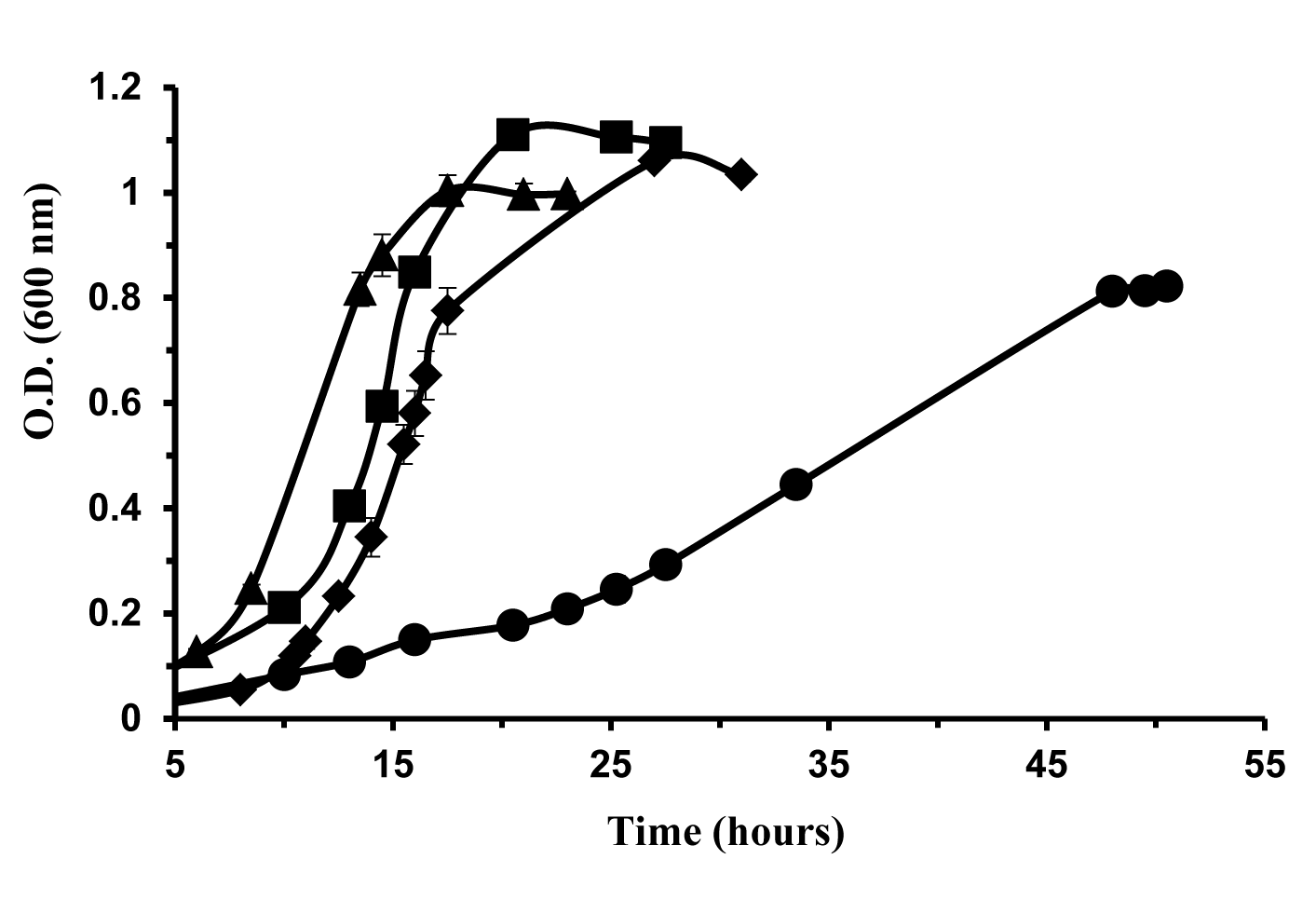

Supplement: Figure S1 [file sph005162167sf4.tif]

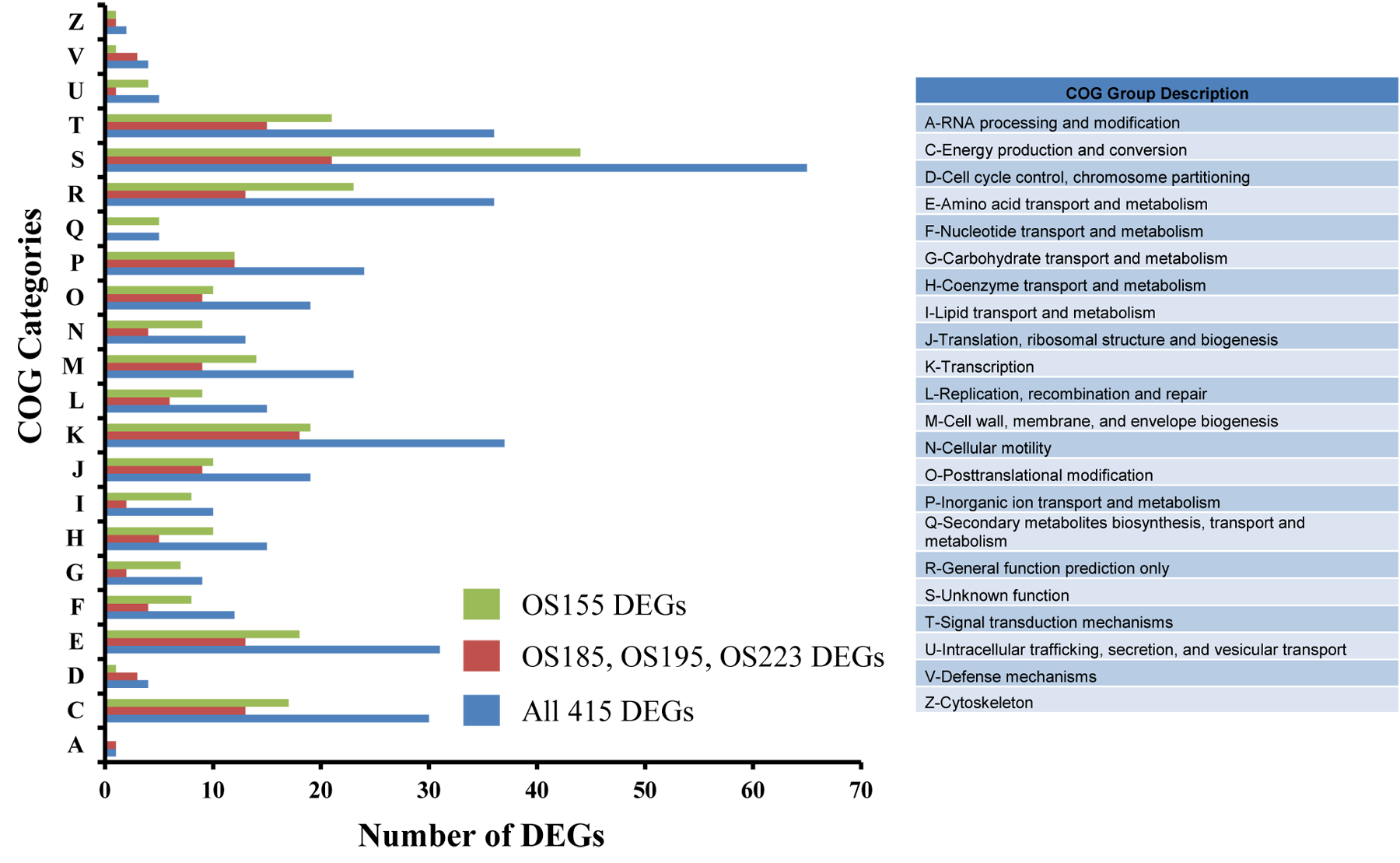

Supplement: Figure S2 [file sph005162167sf5.tif]
