# Supplementary material for: Shewanella baltica Ecotypes Have Wide Transcriptional Variation under the Same Growth Conditions
Source: mSphere. 2016 Oct 19;1(5):e00158-16. doi: 10.1128/mSphere.00158-16 (PMC5071532; doi:10.1128/mSphere.00158-16)
Supplement: Table S1 [file sph005162167st1.doc]

**Supplementary Table S1.** Genome features and gene content of sequenced *Shewanella* *baltica* strains.

| **Strain** | **Genome Size^a^** | **Number of genes^b^** | **Protein coding genes^b^** | **Partially shared genes^c^** | **Strain specific genes^c^** | **NCBI Accession**  **Number** |
| --- | --- | --- | --- | --- | --- | --- |
| OS223 | 5,358,884 | 4,464, [203] | 4,245, [191] | 4,307 (96.5%) | 157 (3.7%) | CP001252 |
| OS195 | 5,547,544 | 4,667, [192] | 4,499, [189] | 4,584 (98.2%) | 84 (1.9%) | CP000891 |
| OS185 | 5,312,910 | 4,521, [75] | 4,323, [71] | 4,467 (98.8%) | 54 (1.2%) | CP000753 |
| OS155 | 5,342,896 | 4,521, [200] | 4,307, [182] | 4,367 (96.6%) | 154 (3.6%) | CP000563 |

^a^ Genome size (bp) was computed as the sum of chromosome and plasmid.

^b^ When applicable, number of genes present in plasmids are shown between brackets.

^c^ Only orthologous genes outside of the core were considered in this analysis.
