# Supplementary material for: Shewanella baltica Ecotypes Have Wide Transcriptional Variation under the Same Growth Conditions
Source: mSphere. 2016 Oct 19;1(5):e00158-16. doi: 10.1128/mSphere.00158-16 (PMC5071532; doi:10.1128/mSphere.00158-16)
Supplement: Table S2 [file sph005162167st2.doc]

**Supplementary Table S2**. Abiotic parameters within the Gotland Deep water column for each strain isolation depth^a^.

| **Strain(s) and Isolation Depth (m)** | | **O_2_ [ml l^-1^]** | **Salinity [%]** | **⁰C** | **H_2_S [µmol l^-1^]** | **N_2_O [nmol l^-1^]** | **NO_3_^-^ [µmol l^-1^]** | **NO_2_^-^ [µmol l^-1^]** | **NH_4_^+^ [µmol l^-1^]** |
| --- | --- | --- | --- | --- | --- | --- | --- | --- | --- |
| OS155 (90) | 1.5 | | 10.0 | 4.0 | 0.0 | 95.0 | 9.0 | 0.3 | 1.0 |
| OS185, OS223 (120) | 1.4 | | 10.5 | 5.0 | 0.0 | 70.0 | 12.0 | 0.0 | 0.0 |
| OS195 (140) | 0.0 | | 11.0 | 5.5 | 2.0 | 20.0 | <0.5 | 0.0 | 10.0 |

^a^ Values extrapolated from Brettar *et. al*. (2001). Measurements were recorded during strain isolation in 1986.
