# Supplementary material for: Shewanella baltica Ecotypes Have Wide Transcriptional Variation under the Same Growth Conditions
Source: mSphere. 2016 Oct 19;1(5):e00158-16. doi: 10.1128/mSphere.00158-16 (PMC5071532; doi:10.1128/mSphere.00158-16)
Supplement: Table S3 [file sph005162167st3.doc]

**Supplementary Table S3.** Primers used for real-time quantitative PCR.

| **Gene** | **Forward (5’-3’)** | **Reverse (5’-3’)** |
| --- | --- | --- |
| *pdxJ* | GCTGCGGTTACTCGTTTAGC | CCTTTGCCATGAGCGTATTT |
| Uridine kinase | AAACAAATGTCCAGCGGTGTGTCC | TCTGGCGAAGCTGTTGATATCCCT |
| *iscS* | GCAGCAGCTTCACCTAAACC | TGTATGGCCCTAAAGGCATC |
| *rpoA* | GAAGCTATCCGTCGTTCTGC | AGGGGTCTTCAGCAACTCAA |
| *gyrB* | TTGCCCGTTTAGAGCAACGCTA | ACGCCACTGCTTTCCATTTCCA |
